# Does multi-disciplinary team (MDT)-working variation impact on cancer patient care experience? Results of a cross-sectional survey in Quebec, Canada

**DOI:** 10.1186/1472-6963-14-S2-P127

**Published:** 2014-07-07

**Authors:** Dominique Tremblay, Danièle Roberge, Nassera Touati, Djamal Berbiche

**Affiliations:** 1 Faculty of Medicine and Health Sciences, Université de Sherbrooke, Longueuil, Québec, Canada; 2 Centre de Recherche Hôpital Charles Le Moyne, Longueil, Québec, Canada; 3 École Nationale d’Administration Publique, Montréal, Québec, Canada

## Background

Multi-disciplinary team (MDT)-working is recognized as a key modality for providing cancer services in the province of Quebec (Canada) and elsewhere [1]. Evidence suggests that the quality of teamworking varies across cancer teams and this may impact on care-providing process, and ultimately on patient care experience [2]. The objective of the study is to evaluate the effects of MDT-working on cancer patients’ perceived experience of care.

## Materials and methods

Data were collected in 2010-11 in 15% of Quebec’s oncology outpatient clinics. Sites (n=9) were purposely selected on the basis of the intensity level of MDT (higher or lower). The sample included 1379 adult cancer patients (response rate 80%). Perceived experience of care was documented by means of a self-administered questionnaire divided into six validated sub-scales: timeliness of services (TIM), communication (COM), patient-centered care (PCC), quality of physical environment (QPE), continuity (CONT) and results of care (RES). Multiple logistic regression models were used to estimate the extent to which patients’ ratings of their care experience differed between levels of MDT-working.

## Results

Patients who were treated in clinics where the MDT-working level is high were 3.99 times (95% CI: 1.89-8.41) more likely to rate positively TIM and also more likely to have a positive opinion of COM (OR: 2.37; 95% CI: 1.25-5.45), of PCC (OR: 2.11; 95% CI: 1.05-4.24) and of CONT (OR: 2.18; 95% CI: 1.07-4.47). Patients’ perception of QPE and RES were not related to the level of MDT-working. Various patients’ characteristics (age, level of education, perceived health status) and organizational attributes (team mandate with regard to oncology services, geographic location, team size) were associated with patients’ ratings of their care experience.

## Conclusions

This study suggests that MDT-working can improve various aspects of perceived patients’ care experience. Significant challenges remain in order to draw clear conclusions about the key elements of MDT-working and its benefits and they will be discussed.
